# Profiling of antibiotic resistance of bacterial species recovered from routine clinical isolates in Ethiopia

**DOI:** 10.1186/s12941-017-0221-1

**Published:** 2017-06-26

**Authors:** Robert-Jan ten Hove, Melaku Tesfaye, Witold Frederik ten Hove, Mesfin Nigussie

**Affiliations:** 1 International Clinical Laboratories, Addis Ababa, Ethiopia; 2 HAN University of Applied Sciences, Nijmegen, The Netherlands

**Keywords:** Antibiogram, Antibiotic, Ethiopia, R, Resistance, Surveillance

## Abstract

**Background:**

With the alarming rise in antibiotic resistance in African countries, the need for a surveillance system in the region has become pressing. The rapid expansion of data networks makes it possible to set up healthcare applications that can be both cost-efficient and effective. Large data sets are available for assessment of current antibiotic resistance among Ethiopian patients. Based on the data-presentation, a practical approach is proposed on how diagnostic laboratories can participate remedial action against antibiotic resistance in Ethiopia.

**Methods:**

In Addis Ababa (Ethiopia), raw data comprising bacterial species name, specimen type and antibiograms covering the period January 2014 to May 2015 was accessed from the laboratory information management system. Using R code, the data was read and fitted into data-frames and analyzed to assess antibiotic resistance in the Ethiopian patient population.

**Results:**

Susceptibility to an antibiotic was tested with 14.983 cultures of 54 different bacterial species or subgroups, isolated from 16 types of specimen. Half of the cultures (n = 6444) showed resistance to an antibiotic. Resistance against penicillin was highest with, on average, 91.1% of 79 bacterial cultures showing resistance. Very high resistance rates were also observed for ampicillin, whereas resistance was lowest with cefoxitin.

**Conclusions:**

Extraction and analysis of raw-data from the laboratory database is relatively simple and can provide valuable insight into the relationships between type of sample and drug-resistance in countries where such data is still scarce. With the largest number of antibiotic resistance tests described for Ethiopia, a tool is proposed for consistent data collection with specified core variables. Trends in antibiotic resistance can be revealed and treatment failures avoided when used as an easy accessible reference application for healthcare providers.

## Background

Irrational use of antibiotics has been perceived to be a major problem worldwide and in particular in the African continent [1–3]. To improve rational drug use in Ethiopia, national standard treatment guidelines were issued to assist health care workers in their treatment of infectious diseases [4]. Treatment guidelines must be continuously revised as susceptibility to antibiotic drugs is constantly threatened due to an empirical approach to treatment and high self-medication of humans and animals without a medical prescription [5, 6]. Enforcing a strict adherence policy in the healthcare sector to reduce the development and spread of drug-resistant bacterial strains goes hand in hand with nationwide antimicrobial surveillance. Routine clinical diagnostic laboratories can contribute to the national surveillance network by sharing routine antibiograms from clinical samples [7, 8]. At the International Clinical Laboratories, routine diagnostic analyses are performed on clinical samples collected at patient service centers across the country and the results are reported back automatically via a virtual private network. The laboratory seeks to extend its services by promoting a healthy life-style to the general public and setting up network applications allowing clinicians to prescribe using mobile networks. Extracting raw data from the laboratory information system and then entering it into a customized data-frame requires some knowledge of programming. In this study, the open source R computing language was used to visualize the prevalence of antibiotic resistance in routine clinical samples in Ethiopia on the basis of raw data extracted from the laboratory information management system, which is the first step towards automated real-time presentation of antibiograms.

## Methods

### Data and sites

Retrospective data from January 2014 to May 2015 was exported from the Polytech laboratory information management system (LIMS) (Comp Pro Med Inc., Santa Rosa, USA) at International Clinical Laboratories, Addis Ababa, Ethiopia. Parameters in the raw data included bacterial species name, type of specimen and antibiotic sensitivity of the bacterial cultures. The data was obtained from routine analysis of clinical specimens collected from individual patients visiting the patient service site, and from clinics and hospitals. All specimens were received and analyzed at the central laboratory in Addis Ababa, Ethiopia.

### Type of specimens

All types of specimen were included in the data query. From patient service sites outside Addis Ababa, only urine specimens were considered feasible for transportation and bacterial analysis at the central laboratory. Specimens that did not match the set categories in the LIMS were renamed as routine samples.

### Bacterial species identification

Preliminary identification was based on number of colonies, types of colonies, morphological appearance and gram staining after growth on both selective and non-selective media. Further bacterial characterization involved biochemical testing and specific growth characteristics under different conditions. All identification tests were carried out according to validated standard operation procedures [9, 10]. After appropriate incubation and based on the site from which the specimen was obtained, the bacterial species were classified as pathogenic or contaminant bacteria. The group of bacteria classified as *Streptococcus* spp. includes all streptococcal species with the exception of the beta-hemolytic streptococcal species, which are categorized as a separate group.

### Antimicrobial susceptibility testing

Antimicrobial susceptibility testing was performed using the Kirby Bauer disk diffusion technique. Standard discs were used to detect and measure induced inhibition for specified antibiotic concentrations, placed on Mueller–Hinton agar supplemented with 5% sheep blood seeded with 0.5 McFarland of bacteria. The plates were incubated overnight under specified conditions (e.g. temperature and atmosphere depending on bacteria species and type of specimen). After incubation was complete, the zone inhibition diameter, in mm, was measured. The zones were interpreted as susceptible, intermediate or resistant [11].

### Quality control

Commercial pre-plated and quality control-passed media were used with 15 different ATCC strains and blank incubation controls to check the transportation and storage conditions of the pre-plated media. The quality control was based on retesting of retained strains and correlation between results of different characteristics of a strain. New batches of ATCC strains for media were checked and compared with previously used ATCC strains and gram stained control slides were used to check the quality of the staining reagents.

### Data analysis

Scripts were written in R (version 3.2.2) to read and fit the raw data into data frames. Test results from *Mycobacterium* cultures were deleted from the data set. The remaining data was summarized and plotted. Anaerobic and aerobic blood cultures were merged in one group. Drug resistance was calculated by dividing the number of resistant cultures by the sum of resistant and sensitive cultures, disregarding the intermediate sensitivity results. The R scripts and raw data are accessible in the Github repository [12].

## Results

Drug susceptibility was tested on 14.983 cultures excluding *Mycobacterium* spp. over the period from January 2014 until May 2015. The total numbers of resistant, intermediate and sensitive cultures were 7440, 1099 and 6444, respectively. Specimens were obtained from urine (n = 11.034), wounds (n = 1346), blood (n = 672), body fluid (n = 466), pediatric blood (n = 411), ear (n = 322), pus (n = 154), stool (n = 136), sputum (n = 134), cerebral spinal fluid (CSF) (n = 94), routine samples (n = 78), eye (n = 45), throat (n = 62), nasal swab (n = 11), nasal discharge (n = 10) and urethral discharge (n = 8) (Table 1).

**Table 1:** Eighteen months of retrospective bacterial analyses showing the most prevalent bacterial species for each sample

Source	Bacterial species most frequently isolated	Frequency of bacterial species	Percentage of bacterial species (%)
Blood	Coagulase-negative *Staphylococcus* species	214	31.8
Blood-pediatric	Coagulase-negative *Staphylococcus* species	157	38.2
Body fluid	*Escherichia coli*	183	39.3
Cerebral spinal fluid	*Staphylococcus aureus*	23	24.5
Ear culture	*Staphylococcus aureus*	79	24.5
Eye culture	*Streptococcus pneumoniae*	26	57.8
Nasal discharge	Group G *Streptococcus*	10	100.0
Nasal swab	Coagulase-negative *Staphylococcus* species	11	100.0
Pus	*Escherichia coli*	73	47.4
Routine	*Escherichia coli*	36	46.2
Sputum	*Klebsiella oxytoca*	49	36.6
Stool	*Salmonella* group non *paratyphi* A/B	43	31.6
Throat	*Streptococcus pyogenes* (Group A)	22	35.5
Urethral discharge	*Escherichia coli*	8	100.0
Urine	*Escherichia coli*	7140	64.7
Wound	*Staphylococcus aureus*	565	36.0

**Fig. 1:**
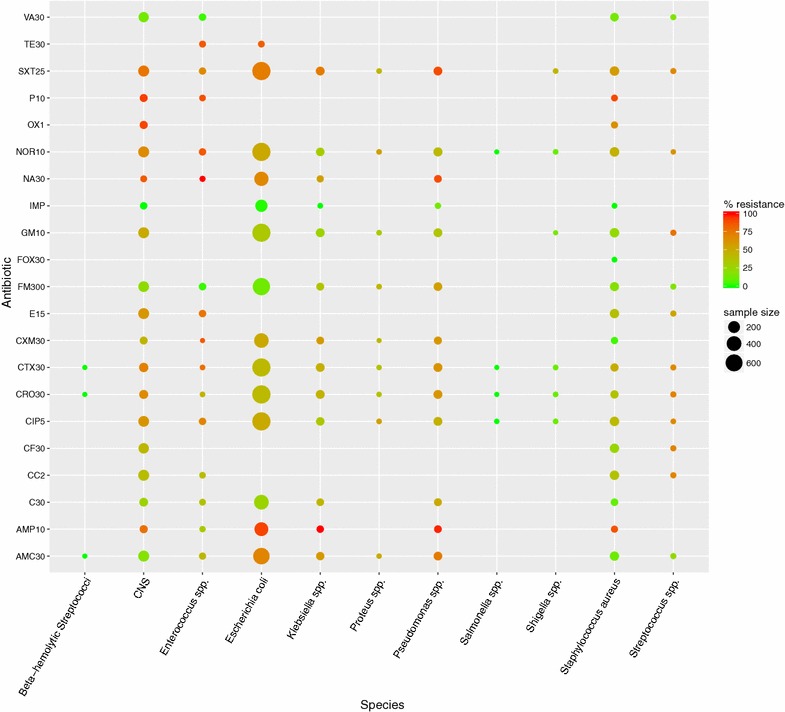
Percentages of resistance of bacterial species (n = 12.220) against antibiotics represented by *color* and number of tested samples represented by *dot-size* (January 2014–May 2015). *CNS* coagulase negative staphylococci, *SAMC30* amoxicillin/clavulanic acid, *AMP10* ampicillin, *C30* chloramphenicol, *CC2* clindamycin, *CF30* cephalophin, *CIP5* ciprofloxacin, *CRO30* ceftriaxone, *CTX30* cefotaxime, *CXM30* cefuroxime, *E15* erythromycin, *FM300* nitrofurantoin, *FOX30* cefoxitin, *GM10* gentamicin, *IMP* imipenem, *NA30* nalidixic acid, *NOR10* norfloxacin, *OX1* oxacillin, *P10* penicillin, *SXT25* sulfamethoxazole, *TE30* tetracycline, *VA30* vancomycin

In total, 54 different bacterial species or subgroups were identified (Appendix 1). To visualize resistance patterns, data was further tailored by merging bacterial species into genus groups. Antibiogram groups containing few cultures and bacterial cultures with intermediate resistance levels were considered not representative of the general antibiotic drug resistance (ADR) profiles and therefore omitted from further analyses. As a result, 1836 cultures were filtered out when the sum of resistant plus sensitive cultures was less than 7 within a group of bacterial species and for a given antibiotic. The remaining 13.147 antibiograms contained 927 intermediate cultures, which were subsequently excluded from the calculation of antibiotic resistance rates. The bacterial antibiotic resistance rate was calculated for each group of bacteria for the 21 different antibiotics (Fig. 1).

The highest resistance rates were observed for penicillin (P10) with on average 91.1% of 79 bacterial cultures showing resistance, with coagulase negative *Staphylococcus* (CNS) (92.9%; n = 42), *Staphylococcus aureus* (90.0%; n = 20) and *Enterococcus* spp. (88.2%; n = 17). High percentages of resistant cultures were also observed for ampicillin (AMP10) with the highest four *Klebsiella* spp. (100%; n = 30), CNS (90.2%; n = 51), *Pseudomonas* spp. (93.3%; n = 60) and *Escherichia coli* (91.5%; n = 331). The lowest resistance rates were observed with cefoxitin in *S. aureus* cultures (FOX30) (0%; n = 10). For imipenem (IMP), no or low resistance rates were observed in cultures with CNS (0%; n = 34), *Klebsiella* spp. (0%; n = 10), *S. aureus* (0%; n = 10), *E. coli* (1.3%, n = 224) and *Pseudomonas* spp. (13.3%; n = 15). Resistance to vancomycin (VA30) was observed with *Enterococcus* spp. (3.3%; n = 30), CNS (13.4%; n = 119), *S. aureus* (14.0%; n = 57) and *Streptococcus* spp. (16.7%; n = 12). A table showing percentages of resistance rates and numbers of cultures from this study is presented in Appendix 2.

## Discussion

This study describes the results from almost fifteen thousand antibiograms analyzed with open-source software; it is the largest number of ADR test results to have been described in Ethiopia. As is the case throughout the rest of Africa, data on antibiotic resistance levels in Ethiopia is minimal and mostly derived from literature. Estimates of ADR percentages are rendered ambiguous as a result of quality assurance issues, small sample sizes and biases related to hospital-based studies with isolates from specific patient groups [6, 13, 14]. Although the interpretation of the data presented in this study must be taken with caution, it does provide a general overview of the current situation, showing similarities with ADR described in other recent studies.

One of the study limitations is the fact that the query in the laboratory database converged one time-period and was restricted to sample source, bacteria species and antibiotic susceptibility result. With the available LIMS query method, it was impossible to distinguish whether multiple specimens originated from one patient. Samples obtained from hospitals might have included patients hospitalized >48 h, which makes it impossible in this study to distinguish between hospital acquired infections and resistance patterns at community level. In addition, because information on antibiotic use prior to the bacterial culture was not available it is not possible to distinguish between patients who had been treated with antibiotics and those who were not. The tradition of empirical antibiotic treatment of presumed infectious illnesses in Ethiopia is such that it can be expected that patients will seek medical advice after treatment failure, which explains the high ADR levels described in this study.

The majority of antibiograms in this study were derived from urine samples in which *E. coli* was most often determined. More than 80% of the cultures showed resistance to ampicillin and tetracycline, while resistance rates to chloramphenicol, nitrofurantoin and imipenem were less than 27%. Overall, the resistance patterns were similar to the results described by Abejew et al. for the Dessie regional health research laboratory [15] in which also high rates was described for ampicillin and tetracycline while susceptible to nitrofurantoin. High rates of multi-drug resistant extended-spectrum beta-lactamase (ESBL) producing enterobacteriaceae uropathogens were described in Gondor hospital [16]. The results of the ESBL tests could not be entered in the LIMS during the study timeframe and were directly reported to physicians. Examination of these reports from the study time also suggests high prevalence of ESBL. After the recent installation of the BD Phoenix 100 instrument at International Clinical Laboratories (ICL) and with the data now being stored in the LIMS, suspected and confirmed ESBL-producing microorganisms are now encountered almost daily (private communication with ICL microbiologist).

The second most numerous specimens received at the laboratory were obtained from wounds, with most infections due to *S. aureus*. These bacteria demonstrated less than 21% resistance to most antibiotics, with the exception of penicillin (n = 20; resistance rate = 90.0%), ampicillin (n = 24; resistance rate = 87.5%) and oxacillin (n = 25; resistance rate = 64.0%). Presence of methicillin-resistant *S. aureus* (MRSA) could not be proven in this study. Other studies indicate high rates of MRSA in the community and amongst health-care workers, however, reliance on phenotypic tests usually provides an overestimation [17–19]. There are no recent estimates of the prevalence of MRSA in the Ethiopian community, therefore, making molecular confirmation methods more accessible in Ethiopia to be crucial for future national surveillance programs.

A comparison of the data obtained with the recommendations in the Ethiopian standard treatment guidelines [4] clearly shows that a high treatment failure rate can be expected when drugs are administered according to national protocols. There are extensive differences in the rates of resistance observed for different bacterial species within one type of specimen. It is therefore important to back up treatment consultations with bacterial species identification accompanied by antibiograms.

Resistance to antibiotics poses serious threats not only in the developing world, but internationally [20]. National and international surveillance initiatives are on-going to identify and publicize trends in resistance rates and, in some instances, have resulted in rapid changes to national treatment guidelines [21, 22]. The crucial ingredient lacking in Ethiopian health policy is the availability of reliable data. The Ethiopia Food, Medicine, Healthcare Administration and Control Authority (EFMHACA) is currently establishing a five strategic plan to decrease antibiotic resistance. This strategy is based on awareness, evidence-based information, infection prevention, optimized use of antimicrobials in human and animal health and strengthening national partnerships. Microbiology laboratories are the first line of identification of antibiotic resistance among the general public. Besides delivering awareness to its clients, the laboratories play a key role in providing evidence-based data for antimicrobial surveillance. Reliable trends are directly correlated with reliable data input. The World Health Organisation published a manual on how to aggregate and validate national ADR files [23]. Additional guidance on how to structure raw data into correct data frames and how to visualize the data into clarifying figures could facilitate and encourage microbiology laboratories to assist in data collection. With currently available information technology, it takes relatively little effort to set up the application with real-time data feed as an additional service from diagnostic laboratories towards all levels: diagnostic laboratories providing the additional service of a real-time data feed that reaches all levels ranging from the clinicians prescribing antibiotics to national surveillance programs.

## Conclusions

The extraction and analysis of raw-data from the laboratory database provides valuable insights into the relationships between type of sample and drug-resistance in countries where such data is still scarce. When used as an easy accessible reference application for healthcare providers, computer-based surveillance can reveal trends in antibiotic resistance levels and thus prevent treatment failures.

## Appendix 1

See Table 2.

**Table 2:** Identified bacteria with frequency in and percentage of the total number of isolates

Bacteria isolated	Frequency	Percentage (%)
*Acinetobacter* species	12	0.08
*Actinomyces viscosus*	9	0.06
*Bacteroides fragilis*	2	0.01
Beta hemolytic non-group A	7	0.05
*Citrobacter braakii*	1844	12.31
*Citrobacter farmeri*	16	0.11
*Citrobacter freundii*	8	0.05
*Citrobacter koseri*	8	0.05
Coagulase-negative *Staphylococcus* species	16	0.11
*Enterobacter cloacae*	59	0.39
*Enterobacter* species	15	0.10
*Enterococcus faecalis*	362	2.42
*Enterococcus* species	12	0.08
*Escherichia coli*	8057	53.77
Group A *Streptococcus*	33	0.22
Group B *Streptococcus*	85	0.57
Group C *Streptococcus*	26	0.17
Group F *Streptococcus*	9	0.06
Group G *Streptococcus*	10	0.07
*Haemophilus influenzae* biotype I	11	0.07
*Haemophilus influenzae*	5	0.03
*Klebsiella ornithinolytica*	36	0.24
*Klebsiella oxytoca*	446	2.98
*Klebsiella pneumoniae*	279	1.86
*Klebsiella* species	54	0.36
*Klebsiella terrigena*	24	0.16
*Moraxella lacunata*	8	0.05
*Morganella morganii*	8	0.05
*Pantoea* species 4 (*Erwina* species)	8	0.05
*Proteus mirabilis*	20	0.13
*Proteus penneri*	8	0.05
*Proteus* species	50	0.33
*Proteus vulgaris*	32	0.21
*Pseudomonas aeruginosa*	154	1.03
*Pseudomonas* species	771	5.15
*Salmonella* group	18	0.12
*Salmonella* paratyphi A	8	0.05
*Salmonella* paratyphi B	11	0.07
*Salmonella* species	41	0.27
*Serratia* species	13	0.09
*Shigella boydii*	19	0.13
*Shigella dysenteriae*	8	0.05
*Shigella* group A1	9	0.06
*Shigella sonnei*	16	0.11
*Shigella* species	22	0.15
*Staphylococcus aureus*	1554	10.37
*Staphylococcus lugdunensis*	294	1.96
*Staphylococcus saprophyticus*	80	0.53
*Streptococcus agalactiae* (group B)	11	0.07
*Streptococcus anginosus/milleri*	21	0.14
*Streptococcus pneumoniae*	63	0.42
*Streptococcus pyogenes* (group A)	76	0.51
*Streptococcus* species	210	1.40
*Viridans streptococci*	5	0.03
Total	14.983	100.00

### Appendix 2

See Table 3.

**Table 3:** Percentage resistance and number of bacterial cultures (in brackets) per antibiotic after merging bacterial species and filtering out antibiogram groups containing less than 7 observations

	β-Hemolytic Streptococci	CNS	*Enterococcus* spp.	*Escherichia coli*	*Klebsiella* spp.	*Proteus* spp.	*Pseudomonas* spp.	*Salmonella* spp.	*Shigella* spp.	*Staphylococcus aureus*	*Streptococcus* spp.
VA30		3.4 (119)	3.3 (30)							3.5 (57)	8.3 (12)
TE30			4.8 (21)	5.3 (19)							
SXT25		2.6 (156)	3.7 (27)	0.7 (740)	4.6 (65)	11.1 (9)	4.6 (65)		11.1 (9)	3.4 (87)	8.3 (12)
P10		4.8 (42)	5.9 (17)							5.0 (20)	
OX1		6.7 (30)								4.0 (25)	
NOR10		2.6 (151)	3.6 (28)	0.7 (726)	3.5 (57)	11.1 (9)	5.4 (74)	14.3 (7)	11.1 (9)	3.4 (89)	12.5 (8)
NA30		5.0 (40)	7.7 (13)	0.6 (363)	4.0 (25)		5.6 (36)				
IMP		6.7 (30)		1.3 (224)	10.0 (10)		6.7 (15)			10.0 (10)	
GM10		2.7 (150)		0.7 (726)	4.5 (66)	11.1 (9)	4.6 (65)		14.3 (7)	3.5 (86)	7.7 (13)
FOX30										10.0 (10)	
FM300		2.8 (143)	3.1 (32)	0.6 (643)	5.1 (39)	11.1 (9)	3.4 (59)			2.7 (73)	8.3 (12)
E15		2.7 (149)	3.4 (29)							3.7 (81)	7.7 (13)
CXM30		5.0 (40)	14.3 (7)	1.0 (388)	5.6 (36)	14.3 (7)	4.7 (43)			3.8 (26)	
CTX30	14.3 (7)	2.4 (82)	11.1 (9)	0.7 (735)	4.6 (65)	12.5 (8)	5.9 (68)	14.3 (7)	11.1 (9)	6.4 (47)	8.3 (12)
CRO30	14.3 (7)	1.4 (71)	11.1 (9)	0.7 (746)	4.5 (67)	12.5 (8)	5.9 (68)	14.3 (7)	11.1 (9)	6.1 (49)	7.7 (13)
CIP5		2.8 (143)	3.4 (29)	0.7 (739)	3.6 (55)	11.1 (9)	4.5 (66)	12.5 (8)	11.1 (9)	3.5 (86)	11.1 (9)
CF30		3.1 (129)								3.6 (83)	7.7 (13)
CC2		2.6 (152)	5.9 (17)							3.6 (84)	7.7 (13)
C30		4.8 (62)	5.3 (19)	1.0 (400)	5.9 (34)		5.0 (40)			6.3 (32)	
AMP10		6.8 (44)	6.7 (15)	0.9 (331)	6.7 (30)		5.6 (36)			4.2 (24)	
AMC30	14.3 (7)	2.6 (152)	4.0 (25)	0.9 (559)	5.9 (51)	12.5 (8)	5.0 (60)			3.7 (3.7)	7.7 (13)

*CNS* coagulase negative staphylococci, *SAMC30* amoxicilline/clavulate acid, *AMP10* ampicillin, *C30* chloroamphenicol, *CC2* clindamycin, *CF30* cephalophin, *CIP5* ciprofloxacin, *CRO30* ceftriaxone, *CTX30* cefotaxime, *CXM30* cefuroxime, *E15* erythromycin, *FM300* nitrofurantoin, *FOX30* cefoxitin, *GM10* gentamicin, *IMP* imipenem, *NA30* nalidixic acid, *NOR10* norfloxacin, *OX1* oxacillin, *P10* penicillin, *SXT25* sulfamethoxazole, *TE30* tetracycline, *VA30* vancomycin

## Abbreviations

ADR: antibiotic drug resistance
ATCC: American Type Culture Collection
CNS: coagulase negative *Staphylococcus*
CSF: cerebral spinal fluid
ESBL: extended-spectrum beta-lactamase
EFMHACA: Ethiopia Food, Medicine, Healthcare Administration and Control Authority
ICL: International Clinical Laboratories
LIMS: laboratory information management system
MRSA: methicillin-resistant *S. aureus*
